# (−)-Istanbulin A

**DOI:** 10.1107/S1600536809033418

**Published:** 2009-09-05

**Authors:** Matías López-Rodríguez, Matías Reina, D. M. Domínguez-Díaz, V. Fajardo, L. Villarroel

**Affiliations:** a Instituto de Bioorgánica "A. González", Universidad de La Laguna, Astrofísico Francisco Sánchez, 2, 38206 La Laguna, Tenerife, Spain; b Instituto de Productos Naturales y Agrobiología (IPNA-CSIC), Astrofísico Francisco Sánchez, 3, 38206 La Laguna, Tenerife, Spain; c Facultad de Ciencias, Universidad de Magallanes (UMAG), Punta Arenas, Chile; d Facultad de Química y Biología, Universidad de Santiago de Chile, Chile

## Abstract

The title compound (systematic name: 9a-hydr­oxy-3,4a,5-trimethyl-4a,6,7,8a,9,9a-hexa­hydro-4*H*,5*H*-naphtho[2,3-*b*]furan-2,8-dione), C_15_H_20_O_4_, is a sesquiterpene lactone showing the typical eremophilanolide skeleton, which has been isolated from the plant *Senecio candidans* collected in the Chilean Magallanes region. The present study confirms the atomic connectivity assigned on the basis of ^1^H and ^13^C NMR spectroscopy, as well as the relative stereochemistry of the 4α-methyl,5α-methyl,8β-hydr­oxy,10β-*H* unit. The crystal structure is stabilized by inter­molecular O—H⋯O hydrogen bonds involving the hydr­oxy group as donor and the oxo group as acceptor, giving chains along the *a* axis. The absolute structure was not determined because of the lack of suitable anomalous scatters.

## Related literature

For the biological activity of metabolites isolated from plants of the *Senecio* species, see: Ulubelen *et al.* (1971 ▶); Burgueño-Tapia *et al.* (2007 ▶); Domínguez *et al.* (2008 ▶); Reina,González-Coloma, Domínguez-Díaz *et al.* (2006 ▶); Reina, González-Coloma, Gutiérrez *et al.* (2006 ▶).
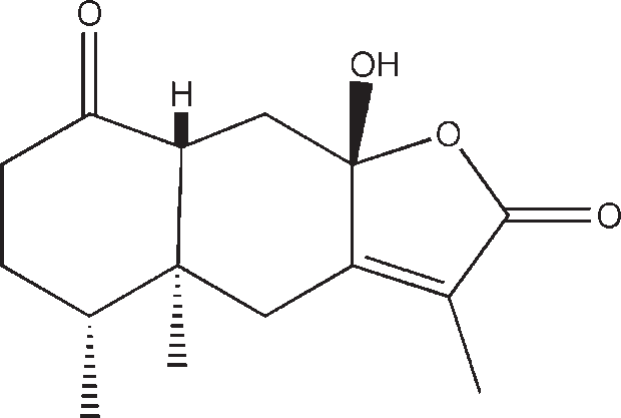


## Experimental

### 

#### Crystal data

C_15_H_20_O_4_
*M* *_r_* = 264.31
Monoclinic, 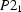

*a* = 7.432 (4) Å
*b* = 13.010 (6) Å
*c* = 8.161 (6) Å
β = 115.47 (4)°
*V* = 712.4 (8) Å^3^
*Z* = 2
Mo *K*α radiation
μ = 0.09 mm^−1^
*T* = 293 K
0.45 × 0.35 × 0.25 mm

#### Data collection

Enraf–Nonius KappaCCD diffractometer
Absorption correction: none
4410 measured reflections
1678 independent reflections
1485 reflections with *I* > 2σ(*I*)
*R*_int_ = 0.018

#### Refinement

*R*[*F* ^2^ > 2σ(*F* ^2^)] = 0.038
*wR*(*F* ^2^) = 0.095
*S* = 1.04
1678 reflections
186 parameters
1 restraint
H atoms treated by a mixture of independent and constrained refinement
Δρ_max_ = 0.20 e Å^−3^
Δρ_min_ = −0.12 e Å^−3^

### 

Data collection: *COLLECT* (Nonius, 2000 ▶); cell refinement: *SCALEPACK* (Otwinowski & Minor, 1997 ▶); data reduction: *SCALEPACK* and *DENZO* (Otwinowski & Minor, 1997 ▶); program(s) used to solve structure: *SIR97* (Altomare *et al.*, 1999 ▶); program(s) used to refine structure: *SHELXL97* (Sheldrick, 2008 ▶ ); molecular graphics: *PLATON* (Spek, 2009 ▶); software used to prepare material for publication: *Win* *GX* (Farrugia,1999 ▶).

## Supplementary Material

Crystal structure: contains datablocks global, I. DOI: 10.1107/S1600536809033418/hg2542sup1.cif

Structure factors: contains datablocks I. DOI: 10.1107/S1600536809033418/hg2542Isup2.hkl

Additional supplementary materials:  crystallographic information; 3D view; checkCIF report

## Figures and Tables

**Table 1:** Hydrogen-bond geometry (Å, °)

*D*—H⋯*A*	*D*—H	H⋯*A*	*D*⋯*A*	*D*—H⋯*A*
O3—H3⋯O4^i^	0.90 (4)	1.87 (4)	2.750 (3)	164 (4)

Symmetry code: (i) 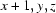.

**Fig. 1.**
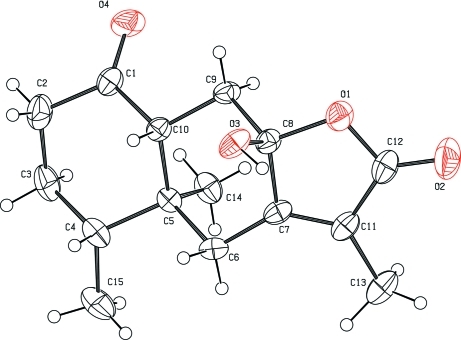
Molecular structure of the title compund showing displacement ellipsoids at the 50% probability level.

**Fig. 2.**
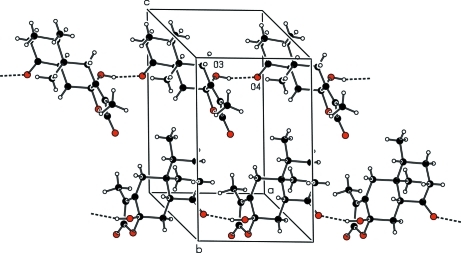
A view of the hydrogen-bonding pattern. Hydrogen atoms not involved in the O—H···O interactions have been omitted.

## supplementary crystallographic information

### Comment

The *Senecio* (Asteraceae) genus are widely distributed by the worldwide and the most studied constituents are sesquiterpenes with eremophilanolides and furanoeremophilanes skeleton, together with pyrrolizidine alkaloids. The chemical study of plants of the *Senecio *species has increased in the last few years because of the biological activity that shown the metabolites isolated from this natural sources: Reina, González-Coloma, Domínguez-Díaz *et al.* (2006); Reina, González-Coloma, Gutiérrez *et al.* (2006); Burgueño-Tapia *et al.* (2007); Domínguez *et al.* (2008).

As part of our ongoing study of *Senecio *genus from the chilean shouthern and Magallanes Region, in this work we report the isolation and the molecular and crystal structure determination of the title compound, which it is described for the first time as a metabolite for the *Senecio* genus. An Istanbulin A with optical rotation [α]_D_^25^ = + 81.5° was reported some years ago and its structure and stereochemistry determined by spectroscopic methods, but no X-ray analysys was performed (Ulubelen *et al.*,1971). The lack of suitable anomalous scatters did not allow us to reliably determine the absolute structure and that shown: 1-oxo-8β-hydroxy-10βH-eremophil-7(11)-en-12,8β-olide was chosen to be, on the basis of the negative optical rotation value: [α]_D_^25^ = - 68.7°, the opposite to that the previously reported compound.

The crystal structure is stabilized by intermolecular O—H···O hydrogen bonds in which are involved the hydroxyl group at C8 acting as donor and the oxo group at C1 as acceptor, giving chains along the *a* axis, through a C(7) graph-set motif.

### Experimental

*Senecio candidans *DC. (Asteraceae) was collected from the south of Chile, at the Santa Maria River, Punta Arenas(XII Region), in april 2002 and authenticated by Professor E. Pisano. A voucher specimen (n° 3483) was deposited in the herbarium of Instituto de la Patagonia, Universidad de Magallanes, Punta Arenas (Chile).

Dried aerial parts of *S.candidans* (3.5 kg) was extracted in methanol at room temperature during a week to give a crude methanolic extract (224 g), 6.4% yield of dry plant weight. A portion of these crude methanolic extract (124 g) was chromatographed on a silica gel vacuum-liquid chromatography column (VLC), using a hexane-ethyl acetate-methanol gradient. The portion of the extract collected at the (hexane-ethyl acetate, 75:25) solvent polarity (2.7 g) was further purified by passage over Sephadex LH-20 (hexane-methylene chloride-methanol, 3:1:1), followed by different chromatographic techniques to give (-)-Istanbuline A: 1-oxo-8β-hydroxy-10βH-eremophil-7(11)-en-12,8β-olide (6.4 mg). The molecular formula C_15_H_20_O_4_ was deduced from its high resolution MS spectrum which shows a molecular ion at *m/z*=264.1357. Complete and unambiguous assignment of de all protons and carbon were established by analysis of the mono and bidimensional NMR experiments and comparison with the previously spectroscopic data reported for Istanbulin A (Ulubelen *et al.*,1971).

The absolute structure was not determined because of the lack of suitable anomalous scatters. However, the value of the measured optical rotation: [α]_D_^25^ = -68.7° allowed to us to choose that shown, as the opposite to that a previously described (+)-istanbulin: [α]_D_^25^ = +81.5°.

### Refinement

All H-atoms were located on sucessive difference-Fourier maps. The H-atom of the hydroxyl group was freely refined. and all other H atoms were constrained refined, with idealized geometries: *C*—H = 0.96(CH_3_), 0.97(CH_2_), 0.98(CH)Å. The lack of suitable anomalous scatters did not allow us to reliably determine the absolute structure and, therefore, the Friedel pairs were merged prior to the final refinement.

### Crystal data

**Table:**

C_15_H_20_O_4_	*F*(000) = 284
*M**_r_* = 264.31	*D*_x_ = 1.232 Mg m^−^^3^
Monoclinic, *P*2_1_	Mo *K*α radiation, λ = 0.71073 Å
Hall symbol: P 2yb	Cell parameters from 297 reflections
*a* = 7.432 (4) Å	θ = 4.3–23.7°
*b* = 13.010 (6) Å	µ = 0.09 mm^−^^1^
*c* = 8.161 (6) Å	*T* = 293 K
β = 115.47 (4)°	Block, colourless
*V* = 712.4 (8) Å^3^	0.45 × 0.35 × 0.25 mm
*Z* = 2	

### Data collection

**Table:**

Enraf–Nonius KappaCCD diffractometer	1485 reflections with *I* > 2σ(*I*)
Radiation source: fine-focus sealed tube	*R*_int_ = 0.018
graphite	θ_max_ = 27.5°, θ_min_ = 6.4°
Detector resolution: 9 pixels mm^-1^	*h* = −9→9
φ and ω scans	*k* = −16→15
4410 measured reflections	*l* = −10→10
1678 independent reflections	

### Refinement

**Table:**

Refinement on *F*^2^	Primary atom site location: structure-invariant direct methods
Least-squares matrix: full	Secondary atom site location: difference Fourier map
*R*[*F*^2^ > 2σ(*F*^2^)] = 0.038	Hydrogen site location: difference Fourier map
*wR*(*F*^2^) = 0.095	H atoms treated by a mixture of independent and constrained refinement
*S* = 1.04	*w* = 1/[σ^2^(*F*_o_^2^) + (0.0482*P*)^2^ + 0.0898*P*] where *P* = (*F*_o_^2^ + 2*F*_c_^2^)/3
1678 reflections	(Δ/σ)_max_ = 0.001
186 parameters	Δρ_max_ = 0.20 e Å^−^^3^
1 restraint	Δρ_min_ = −0.11 e Å^−^^3^

### Special details

**Table:**

Geometry. All e.s.d.'s (except the e.s.d. in the dihedral angle between two l.s. planes) are estimated using the full covariance matrix. The cell e.s.d.'s are taken into account individually in the estimation of e.s.d.'s in distances, angles and torsion angles; correlations between e.s.d.'s in cell parameters are only used when they are defined by crystal symmetry. An approximate (isotropic) treatment of cell e.s.d.'s is used for estimating e.s.d.'s involving l.s. planes.
Refinement. Refinement of *F*^2^ against ALL reflections. The weighted *R*-factor *wR* and goodness of fit *S* are based on *F*^2^, conventional *R*-factors *R* are based on *F*, with *F* set to zero for negative *F*^2^. The threshold expression of *F*^2^ > σ(*F*^2^) is used only for calculating *R*-factors(gt) *etc*. and is not relevant to the choice of reflections for refinement. *R*-factors based on *F*^2^ are statistically about twice as large as those based on *F*, and *R*- factors based on ALL data will be even larger.

### Fractional atomic coordinates and isotropic or equivalent isotropic displacement parameters (Å^2^)

**Table:**

	*x*	*y*	*z*	*U*_iso_*/*U*_eq_	
O1	0.4955 (3)	0.50383 (14)	0.8319 (2)	0.0568 (5)	
O2	0.5421 (4)	0.5731 (2)	0.6018 (3)	0.0869 (7)	
O3	0.6082 (2)	0.37119 (15)	1.04064 (19)	0.0521 (4)	
H3	0.713 (5)	0.354 (3)	1.018 (4)	0.079 (9)*	
O4	−0.0560 (2)	0.35876 (16)	0.9801 (3)	0.0635 (5)	
C1	0.0324 (3)	0.28290 (19)	0.9675 (3)	0.0456 (5)	
C2	−0.0216 (4)	0.1763 (2)	0.9971 (4)	0.0635 (7)	
H2A	−0.1422	0.1778	1.0153	0.079 (9)*	
H2B	0.0844	0.1476	1.1053	0.073 (9)*	
C3	−0.0542 (4)	0.1087 (2)	0.8333 (4)	0.0643 (7)	
H3A	−0.1759	0.1299	0.7312	0.074 (9)*	
H3B	−0.0713	0.0379	0.8615	0.059 (8)*	
C4	0.1189 (4)	0.11441 (18)	0.7792 (3)	0.0518 (6)	
H4	0.2377	0.0891	0.8827	0.059 (7)*	
C5	0.1632 (3)	0.22659 (16)	0.7435 (3)	0.0375 (4)	
C6	0.3567 (3)	0.22904 (18)	0.7137 (3)	0.0448 (5)	
H6A	0.4602	0.1896	0.8084	0.057 (7)*	
H6B	0.3309	0.1977	0.5979	0.050 (7)*	
C7	0.4259 (3)	0.33673 (18)	0.7168 (3)	0.0422 (5)	
C8	0.4541 (3)	0.40177 (17)	0.8797 (3)	0.0412 (5)	
C9	0.2648 (3)	0.40183 (17)	0.9078 (3)	0.0407 (5)	
H9A	0.1589	0.4368	0.8076	0.036 (5)*	
H9B	0.2875	0.4377	1.0192	0.043 (6)*	
C10	0.2059 (3)	0.29068 (16)	0.9181 (3)	0.0372 (4)	
H10	0.3200	0.2583	1.0170	0.044 (6)*	
C11	0.4554 (3)	0.3931 (2)	0.5961 (3)	0.0488 (6)	
C12	0.5040 (4)	0.4988 (3)	0.6676 (4)	0.0588 (6)	
C13	0.4395 (4)	0.3664 (3)	0.4119 (3)	0.0668 (8)	
H13A	0.5521	0.3940	0.3982	0.090*	
H13B	0.3190	0.3950	0.3207	0.090*	
H13C	0.4374	0.2930	0.3988	0.090*	
C14	−0.0102 (3)	0.27228 (19)	0.5782 (3)	0.0512 (5)	
H14A	0.0173	0.3431	0.5648	0.090*	
H14B	−0.1303	0.2676	0.5947	0.090*	
H14C	−0.0266	0.2348	0.4713	0.090*	
C15	0.0800 (5)	0.0415 (2)	0.6210 (5)	0.0771 (9)	
H15A	0.1886	0.0454	0.5872	0.090*	
H15B	−0.0415	0.0607	0.5195	0.090*	
H15C	0.0685	−0.0276	0.6569	0.090*	

### Atomic displacement parameters (Å^2^)

**Table:**

	*U*^11^	*U*^22^	*U*^33^	*U*^12^	*U*^13^	*U*^23^
O1	0.0612 (10)	0.0542 (10)	0.0633 (10)	−0.0224 (8)	0.0348 (8)	−0.0122 (8)
O2	0.0968 (16)	0.0837 (16)	0.0944 (16)	−0.0249 (13)	0.0548 (14)	0.0156 (13)
O3	0.0339 (7)	0.0817 (12)	0.0434 (7)	−0.0078 (8)	0.0194 (6)	−0.0091 (8)
O4	0.0465 (9)	0.0680 (12)	0.0928 (12)	−0.0034 (9)	0.0460 (9)	−0.0125 (10)
C1	0.0355 (10)	0.0562 (14)	0.0480 (11)	−0.0048 (10)	0.0208 (9)	−0.0015 (11)
C2	0.0559 (14)	0.0680 (18)	0.0766 (17)	−0.0052 (13)	0.0378 (13)	0.0159 (15)
C3	0.0604 (15)	0.0452 (14)	0.0848 (18)	−0.0128 (12)	0.0289 (14)	0.0092 (14)
C4	0.0517 (12)	0.0354 (11)	0.0592 (13)	0.0019 (10)	0.0153 (11)	0.0043 (11)
C5	0.0343 (9)	0.0339 (10)	0.0398 (9)	0.0004 (8)	0.0118 (8)	−0.0005 (9)
C6	0.0463 (11)	0.0476 (12)	0.0440 (10)	0.0054 (10)	0.0228 (9)	−0.0063 (10)
C7	0.0325 (9)	0.0558 (13)	0.0408 (10)	−0.0020 (9)	0.0183 (8)	−0.0071 (10)
C8	0.0384 (10)	0.0472 (12)	0.0423 (10)	−0.0099 (9)	0.0213 (8)	−0.0077 (9)
C9	0.0367 (9)	0.0430 (12)	0.0488 (11)	−0.0050 (8)	0.0244 (9)	−0.0109 (9)
C10	0.0282 (8)	0.0438 (11)	0.0407 (10)	0.0015 (8)	0.0161 (7)	0.0002 (9)
C11	0.0378 (10)	0.0703 (16)	0.0414 (10)	−0.0015 (10)	0.0201 (8)	0.0010 (11)
C12	0.0495 (13)	0.0745 (18)	0.0584 (13)	−0.0133 (12)	0.0290 (11)	0.0039 (13)
C13	0.0641 (14)	0.099 (2)	0.0423 (11)	−0.0002 (16)	0.0278 (11)	0.0043 (14)
C14	0.0465 (12)	0.0464 (13)	0.0465 (11)	0.0016 (10)	0.0066 (9)	0.0057 (11)
C15	0.089 (2)	0.0412 (14)	0.093 (2)	−0.0098 (14)	0.0316 (18)	−0.0157 (14)

### Geometric parameters (Å, °)

**Table:**

O1—C12	1.371 (3)	C6—H6A	0.9700
O1—C8	1.454 (3)	C6—H6B	0.9700
O2—C12	1.198 (4)	C7—C11	1.320 (3)
O3—C8	1.379 (3)	C7—C8	1.512 (3)
O3—H3	0.90 (4)	C8—C9	1.519 (3)
O4—C1	1.214 (3)	C9—C10	1.524 (3)
C1—C2	1.493 (4)	C9—H9A	0.9700
C1—C10	1.511 (3)	C9—H9B	0.9700
C2—C3	1.530 (4)	C10—H10	0.9800
C2—H2A	0.9700	C11—C12	1.477 (4)
C2—H2B	0.9700	C11—C13	1.497 (3)
C3—C4	1.532 (4)	C13—H13A	0.9600
C3—H3A	0.9700	C13—H13B	0.9600
C3—H3B	0.9700	C13—H13C	0.9600
C4—C15	1.526 (4)	C14—H14A	0.9600
C4—C5	1.551 (3)	C14—H14B	0.9600
C4—H4	0.9800	C14—H14C	0.9600
C5—C14	1.529 (3)	C15—H15A	0.9600
C5—C6	1.558 (3)	C15—H15B	0.9600
C5—C10	1.561 (3)	C15—H15C	0.9600
C6—C7	1.489 (3)		
			
C12—O1—C8	108.86 (19)	O1—C8—C7	103.91 (17)
C8—O3—H3	108 (2)	O3—C8—C9	107.47 (17)
O4—C1—C2	123.30 (19)	O1—C8—C9	110.88 (18)
O4—C1—C10	121.5 (2)	C7—C8—C9	110.20 (16)
C2—C1—C10	115.2 (2)	C8—C9—C10	108.33 (16)
C1—C2—C3	110.2 (2)	C8—C9—H9A	110.0
C1—C2—H2A	109.6	C10—C9—H9A	110.0
C3—C2—H2A	109.6	C8—C9—H9B	110.0
C1—C2—H2B	109.6	C10—C9—H9B	110.0
C3—C2—H2B	109.6	H9A—C9—H9B	108.4
H2A—C2—H2B	108.1	C1—C10—C9	112.13 (17)
C2—C3—C4	112.8 (2)	C1—C10—C5	110.24 (17)
C2—C3—H3A	109.0	C9—C10—C5	114.02 (16)
C4—C3—H3A	109.0	C1—C10—H10	106.7
C2—C3—H3B	109.0	C9—C10—H10	106.7
C4—C3—H3B	109.0	C5—C10—H10	106.7
H3A—C3—H3B	107.8	C7—C11—C12	108.2 (2)
C15—C4—C3	109.7 (2)	C7—C11—C13	130.8 (3)
C15—C4—C5	113.8 (2)	C12—C11—C13	120.9 (2)
C3—C4—C5	111.89 (19)	O2—C12—O1	121.3 (3)
C15—C4—H4	107.0	O2—C12—C11	129.7 (2)
C3—C4—H4	107.0	O1—C12—C11	108.9 (2)
C5—C4—H4	107.0	C11—C13—H13A	109.5
C14—C5—C4	111.40 (18)	C11—C13—H13B	109.5
C14—C5—C6	109.80 (18)	H13A—C13—H13B	109.5
C4—C5—C6	109.48 (18)	C11—C13—H13C	109.5
C14—C5—C10	111.15 (18)	H13A—C13—H13C	109.5
C4—C5—C10	107.89 (17)	H13B—C13—H13C	109.5
C6—C5—C10	106.99 (16)	C5—C14—H14A	109.5
C7—C6—C5	110.60 (17)	C5—C14—H14B	109.5
C7—C6—H6A	109.5	H14A—C14—H14B	109.5
C5—C6—H6A	109.5	C5—C14—H14C	109.5
C7—C6—H6B	109.5	H14A—C14—H14C	109.5
C5—C6—H6B	109.5	H14B—C14—H14C	109.5
H6A—C6—H6B	108.1	C4—C15—H15A	109.5
C11—C7—C6	132.6 (2)	C4—C15—H15B	109.5
C11—C7—C8	109.9 (2)	H15A—C15—H15B	109.5
C6—C7—C8	117.24 (18)	C4—C15—H15C	109.5
O3—C8—O1	109.53 (17)	H15A—C15—H15C	109.5
O3—C8—C7	114.85 (19)	H15B—C15—H15C	109.5
			
O4—C1—C2—C3	126.2 (3)	O3—C8—C9—C10	−72.0 (2)
C10—C1—C2—C3	−53.2 (3)	O1—C8—C9—C10	168.34 (16)
C1—C2—C3—C4	51.1 (3)	C7—C8—C9—C10	53.8 (2)
C2—C3—C4—C15	177.4 (2)	O4—C1—C10—C9	6.0 (3)
C2—C3—C4—C5	−55.3 (3)	C2—C1—C10—C9	−174.5 (2)
C15—C4—C5—C14	59.4 (3)	O4—C1—C10—C5	−122.1 (2)
C3—C4—C5—C14	−65.6 (3)	C2—C1—C10—C5	57.3 (2)
C15—C4—C5—C6	−62.2 (3)	C8—C9—C10—C1	173.39 (16)
C3—C4—C5—C6	172.73 (19)	C8—C9—C10—C5	−60.4 (2)
C15—C4—C5—C10	−178.3 (2)	C14—C5—C10—C1	66.0 (2)
C3—C4—C5—C10	56.6 (2)	C4—C5—C10—C1	−56.4 (2)
C14—C5—C6—C7	69.0 (2)	C6—C5—C10—C1	−174.11 (18)
C4—C5—C6—C7	−168.42 (18)	C14—C5—C10—C9	−61.1 (2)
C10—C5—C6—C7	−51.8 (2)	C4—C5—C10—C9	176.45 (16)
C5—C6—C7—C11	−120.6 (2)	C6—C5—C10—C9	58.7 (2)
C5—C6—C7—C8	53.2 (2)	C6—C7—C11—C12	173.9 (2)
C12—O1—C8—O3	119.6 (2)	C8—C7—C11—C12	−0.3 (2)
C12—O1—C8—C7	−3.5 (2)	C6—C7—C11—C13	−3.7 (4)
C12—O1—C8—C9	−121.9 (2)	C8—C7—C11—C13	−177.9 (2)
C11—C7—C8—O3	−117.3 (2)	C8—O1—C12—O2	−177.8 (2)
C6—C7—C8—O3	67.5 (2)	C8—O1—C12—C11	3.5 (3)
C11—C7—C8—O1	2.3 (2)	C7—C11—C12—O2	179.5 (3)
C6—C7—C8—O1	−172.85 (17)	C13—C11—C12—O2	−2.6 (4)
C11—C7—C8—C9	121.1 (2)	C7—C11—C12—O1	−2.0 (3)
C6—C7—C8—C9	−54.0 (2)	C13—C11—C12—O1	175.8 (2)

### Hydrogen-bond geometry (Å, °)

**Table:**

*D*—H···*A*	*D*—H	H···*A*	*D*···*A*	*D*—H···*A*
O3—H3···O4^i^	0.90 (4)	1.87 (4)	2.750 (3)	164 (4)

Symmetry codes: (i) *x*+1, *y*, *z*.
